# Assessment of the Prevalence and Types of Mental Compulsions in Patients With Obsessive-Compulsive Disorder in North Karnataka: A Cross-Sectional Study

**DOI:** 10.7759/cureus.71960

**Published:** 2024-10-20

**Authors:** Varinder Pal, Santosh Ramdurg, Shivakumar Chaukimath

**Affiliations:** 1 Psychiatry, Shri B. M. Patil Medical College Hospital and Research Centre, Bijapur Lingayat District Education (BLDE) (Deemed to be University), Vijayapura, IND

**Keywords:** mental checking, mental compulsions, mental compulsions checklist, mental compulsions prevalence, mental rituals, obsessive-compulsive disorder (ocd), religious obsessions, sexual obsessions

## Abstract

Background: Obsessive-compulsive disorder (OCD) is a significant mental condition characterized by the presence of obsessions and compulsions. Mental compulsions are defined as compulsions with no overt behavioral or motoric symptoms, such as repeating certain words or phrases in the mind. The exact prevalence of mental compulsions could be underestimated due to measurement issues using the Y-BOCS (Yale-Brown Obsessive Compulsive Scale) and its clinical characteristics. They are a more chronic and severe form of OCD and respond poorly to cognitive behavioral therapy. Given the relatively high prevalence of mental compulsions, the limited research on their phenomenology and clinical correlations, and the potentially significant implications for treatment, further research is needed.

Methodology: This cross-sectional study was carried out after obtaining the institutional ethical committee clearance of Bijapur Lingayat District Education (BLDE) (Deemed to be University) Shri B.M. Patil Medical College Hospital and Research Centre, Vijayapura. People in the age group of 10 to 60 years with a diagnosis of OCD were assessed for symptom profiles using the dimensional Y-BOCS (DY-BOCS) and a mental compulsions checklist.

Results: In a sample of 80 participants with OCD, the overall point prevalence of mental compulsions in patients with OCD was 53.75% (n=43). Most of these patients had behavioral compulsions as well. Among 43 patients having mental compulsions, obsessions about sacrilege and blasphemy were most common (35%), followed by obsessions related to symmetry (32%). The most common mental compulsions were undoing "bad" thoughts with "good" thoughts (51%), followed by praying (40%) and reassuring themselves (32%).

Conclusions: Mental compulsions were present in over 50% of the study population. They were the most prevalent, following religious, symmetry, and harm-related obsessions. Most of the patients had multiple mental compulsions. The most common types were undoing bad thoughts with good thoughts, followed by praying, reassuring themselves, and repeating phrases or mantras.

## Introduction

Obsessive-compulsive disorder (OCD) is a significant mental health disorder because of its frequency, the disability it causes, and the fact that it is a prime example of the group of disorders known as obsessive-compulsive and related disorders. As per the International Classification of Diseases, 11th edition (ICD-11), OCD is defined by the presence of obsessions and compulsions. Obsessions are repetitive and persistent thoughts, images, impulses, or urges that are intrusive, unwanted, and often associated with anxiety. Compulsions are repetitive behaviors, including repetitive mental acts that the individual feels driven to perform in response to an obsession, according to rigid rules, or to achieve a sense of "completeness." Mental compulsions are defined as compulsions performed by the person in their mind and have no overt behavioral or motoric symptoms. Examples include repeating certain words or phrases in the mind, praying, counting words, letters, or numbers, mental checking, and neutralizing disturbing mental pictures by replacement with another image or thought [1].

Despite the inclusion of mental compulsions in the Diagnostic and Statistical Manual of Mental Disorders-IV (DSM-IV) diagnostic criteria, few studies have explored their phenomenology and clinical correlates. The available data suggest that mental compulsions are common in people with OCD, with a prevalence of 9.8% to 25% in individuals with OCD, with some studies estimating a prevalence of up to 60% of patients [2]. A multicentric study found the prevalence of mental compulsions in patients with OCD to be 56.7% [3]. Another recent study in OCD showed a lifetime prevalence of mental compulsions to be 55.4% [4].

There are several reasons why the exact prevalence of mental compulsions may be understated. For example, Abramowitz et al. [2] highlighted measurement issues and incomplete symptom analysis in studies using the Yale-Brown Obsessive-Compulsive Scale (Y-BOCS) and Symptom Checklist [5]. These tools include mental compulsions and other items in the "miscellaneous compulsions" category, which are more often than not removed from data analyses or treated as a single item, preventing the assessment of their prevalence and clinical characteristics.

Aside from measuring difficulties, clinical considerations may influence reporting. Studies have shown that mental compulsions are common following sexual, repulsive, aggressive, or blasphemous obsessions [6,7], which tend to be stigmatizing, making identification of this type of OCD difficult. The person may be silently repeating words or phrases in their head, praying, mentally counting until they feel "right," mentally checking, replaying memories or conversations, and replacing "bad" thoughts with "good" emotions. As these mental compulsions can be performed quickly and in any place without others perceiving them, they become more difficult to resist and manage. Researchers surmise that patients' hesitation to reveal and confront these thoughts during therapy may stem from the emotional and moral weight of their thoughts, as well as their fear of the healthcare provider misinterpreting them [8]. Research indicates that individuals whose primary compulsion is mental face unique challenges in managing their compulsive behaviors. Mental compulsions, often used as a form of cognitive avoidance during exposure and response prevention, hinder the patient's ability to acclimate to anxiety-inducing stimuli in therapy or daily life [9]. This underscores the need for a more nuanced approach to treatment for these individuals.

This study seeks to fill critical gaps in the research on OCD by focusing on the underexplored area of mental compulsions, especially within an Indian population. This research could provide valuable insights into the prevalence, phenomenology, and clinical implications of mental compulsions, potentially leading to more effective and individualized treatment approaches. As mental compulsions are often overlooked in the broader OCD literature, this study is poised to contribute significantly to the global understanding of the disorder and help bridge existing research gaps.

## Materials and methods

Study design

This is a cross-sectional, interview-based study of a total of 80 study participants who were diagnosed with OCD as per ICD-11. We included all the participants with a primary diagnosis of OCD who visited the outpatient department from August 2022 to February 2024.

Sample size collection

The study needed a sample size of 80 to reach a power of 80%, a two-sided p-value of 0.05, and an effect size of 0.094 using G* Power software 3.1.9.7 (Heinrich Heine University Düsseldorf, Düsseldorf, Germany). The anticipated proportion of OCD was 0.6% [10].

Inclusion and exclusion criteria

We included the patients with a primary diagnosis of OCD as per ICD-11 between the age groups of 10 and 60 years. Patients with schizophrenia, any other psychotic illness, or having an intellectual disability were excluded because these patients can find it challenging to articulate their obsessive-compulsive symptoms, which occasionally may be caused by antipsychotics and make diagnosis more difficult.

Methods

We obtained informed written consent from the patients before the sample collection. In the case of patients aged <18 years, we obtained informed consent from the parents/guardians. Approval from the ethics committee of our institution (Institutional Review Board) was taken. We recorded the participants' replies on a proforma that included demographic information such as their initials, age, religion, residence, occupation, education, onset of symptoms, and age at first visit to the hospital for the OCD symptoms. We used the 11th revision of the ICD to diagnose OCD in every participant during the interview. The diagnosis was made using a semi-structured interview by the Senior Consultant Psychiatrist of the department. The research team was different from the treating team. We also applied the Dimensional Yale-Brown Obsessive-Compulsive Scale Symptom Checklist (DY-BOCS) [11] to evaluate OCD symptoms according to eight dimensions. It provides both obsessions and compulsions, both behavioral and mental, within each dimension. DY-BOCS was used instead of the Y-BOCS symptom checklist as it asks about mental compulsions inside each symptom dimension.

We developed a Mental Compulsions Checklist based on the most common types of mental compulsions observed in the literature, which included (1) praying, (2) undoing bad thoughts with good thoughts, (3) repeating phrases/mantras, (4) reassuring themselves, (5) organizing objects mentally or mental checking, (6) counting/numbers mentally, and (7) others.

Statistical analysis

The acquired data were entered into a Microsoft Excel (Microsoft Corporation, Redmond, Washington) document, and IBM SPSS Statistics for Windows, Version 27 (Released 2020; IBM Corp., Armonk, New York) was used for statistical analysis. The findings were displayed using charts, percentages, mean±SD, median, interquartile range, and frequency.

## Results

Of the 80 patients, 51.2% (n=41) were females, and 48.8% (n=39) were males; 58.75% (n=47) of the sample was aged less than 30 years, 60% (n=48) had studied above high school, and 56.3% (n=45) were married. The sociodemographic profile of the final sample is given in Table 1.

**Table 1:** Sociodemographic profile

Parameter	Frequency	Percentage
Age	-	-
<20	9	11.3
20-29	38	47.5
30-39	23	28.7
40-49	7	8.8
50-59	3	3.8
Sex	-	-
Male	39	48.8
Female	41	51.2
Education	-	-
Illiterate	9	11.3
Up to middle school	3	3.8
Middle to high school	20	25.0
Precollege	18	22.5
Graduate	30	37.5
Occupation	-	-
Student	23	28.7
Housewife	19	23.8
Service	11	13.8
Farmer	8	10.0
Unemployed	8	10.0
Business	6	7.5
Daily wage worker	5	6.3
Marital status	-	-
Unmarried	33	41.3
Married	45	56.3
Divorced/widowed	2	2.6
Religion	-	-
Hindu	53	66.3
Muslim	27	33.8
Socioeconomic status	-	-
Lower socioeconomic status	36	45.0
Middle socioeconomic status	27	33.8
Upper socioeconomic status	17	21.3

The mean age of onset of symptoms was 22.32 years (SD=5.6), where the mean age of onset of symptoms in males was 21.2 years (SD=4.4) and in females was 23.4 years (SD=6.3).

Prevalence of mental compulsions

**Figure 1:**
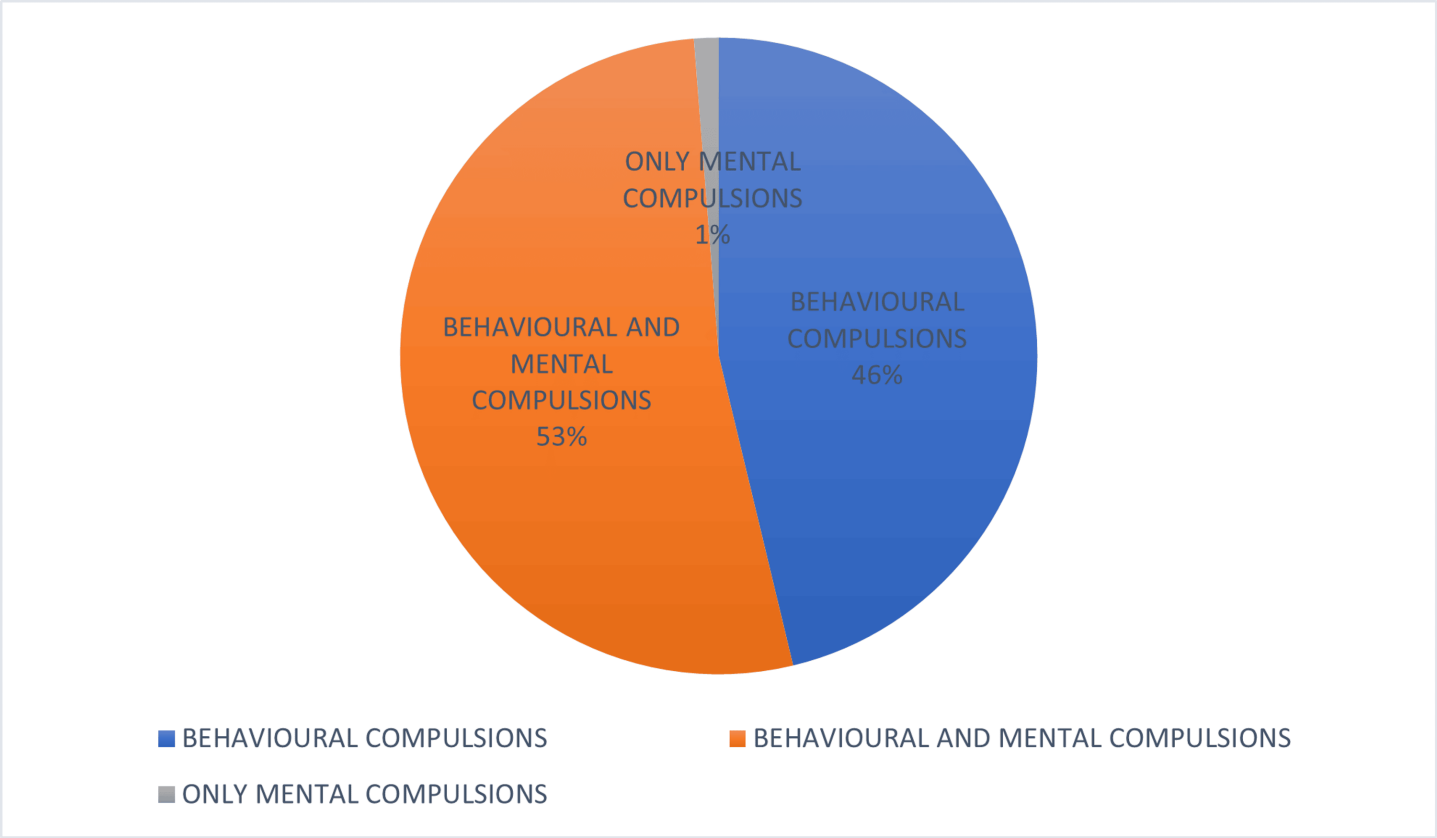
Types of compulsions

The overall point prevalence of mental compulsions in patients with OCD was 53.75% (n=43). Most of these patients had behavioral compulsions as well; 53% of the subjects had mental compulsions and behavioral compulsions, while 46% had only behavioral compulsions, and only 1% had only mental compulsions (Figure 1). Mental compulsions were more common in males (58.13%, n=25) than in females (41.87%, n=18), although this was not statistically significant (chi-square: 3.281, p=0.070). The prevalence of mental compulsions is given in Table 2.

**Table 2:** Prevalence of mental compulsions

Parameter	Frequency	Percentage
Overall	43	53.75
Sex		
Male	25	58.13
Female	18	41.87
Age		
<20	4	9.30
20-29	23	53.48
30-39	14	32.55
40-49	2	4.65
Education		
Illiterate	6	13.95
Up to middle school	1	2.32
Middle to high school	9	20.93
Precollege	10	23.25
College	17	39.53
Occupation		
Student	11	25.58
Housewife	8	18.60
Service	6	13.95
Farmer	6	13.95
Unemployed	3	6.97
Business	4	9.30
Daily wage worker	5	11.62
Marital status		
Married	27	62.79
Unmarried	14	32.55
Divorced/widowed	2	4.65
Religion		
Hindu	30	69.76
Muslim	13	30.23
Socioeconomic status		
Lower socioeconomic status	21	48.83
Middle socioeconomic status	11	25.58
Upper socioeconomic status	11	25.58

Most participants endorsed multiple compulsions, i.e., approximately 70% of participants had more than one mental compulsion. In total, 10 out of 80 patients (12.5%) had mental compulsions as a primary compulsion in one of the domains. Out of 10, five had primary mental compulsions for harm obsessions, and five had primary mental compulsions for religious and sexual obsessions. Only one patient had only mental compulsions and no behavioral compulsion, and it was secondary to an obsession that she might harm someone without meaning to harm them or blurt insults to someone. She had mental compulsions in the form of replacing bad thoughts with good thoughts and reassuring herself.

Types of mental compulsions

**Figure 2:**
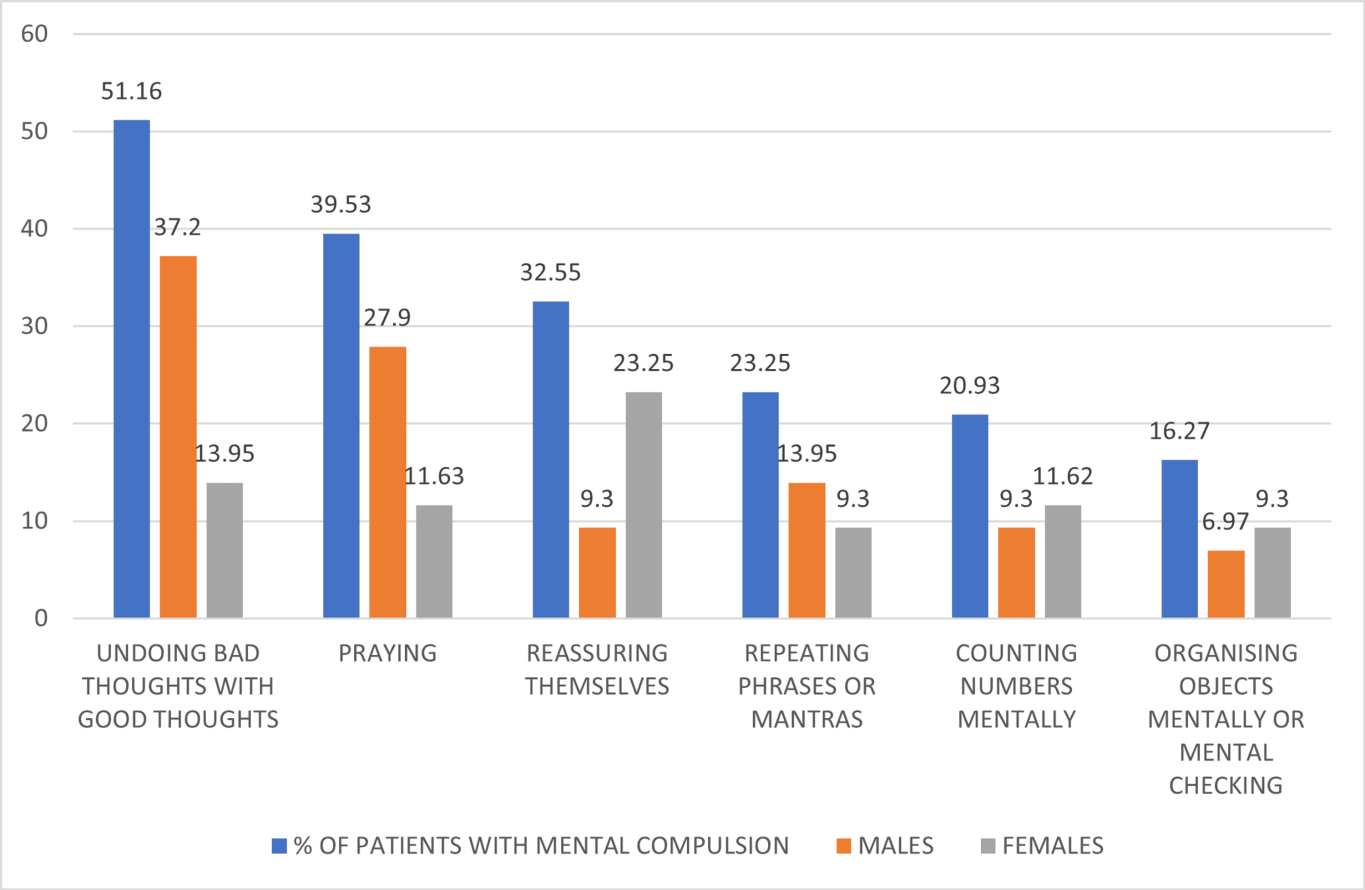
Types of mental compulsions

**Figure 3:**
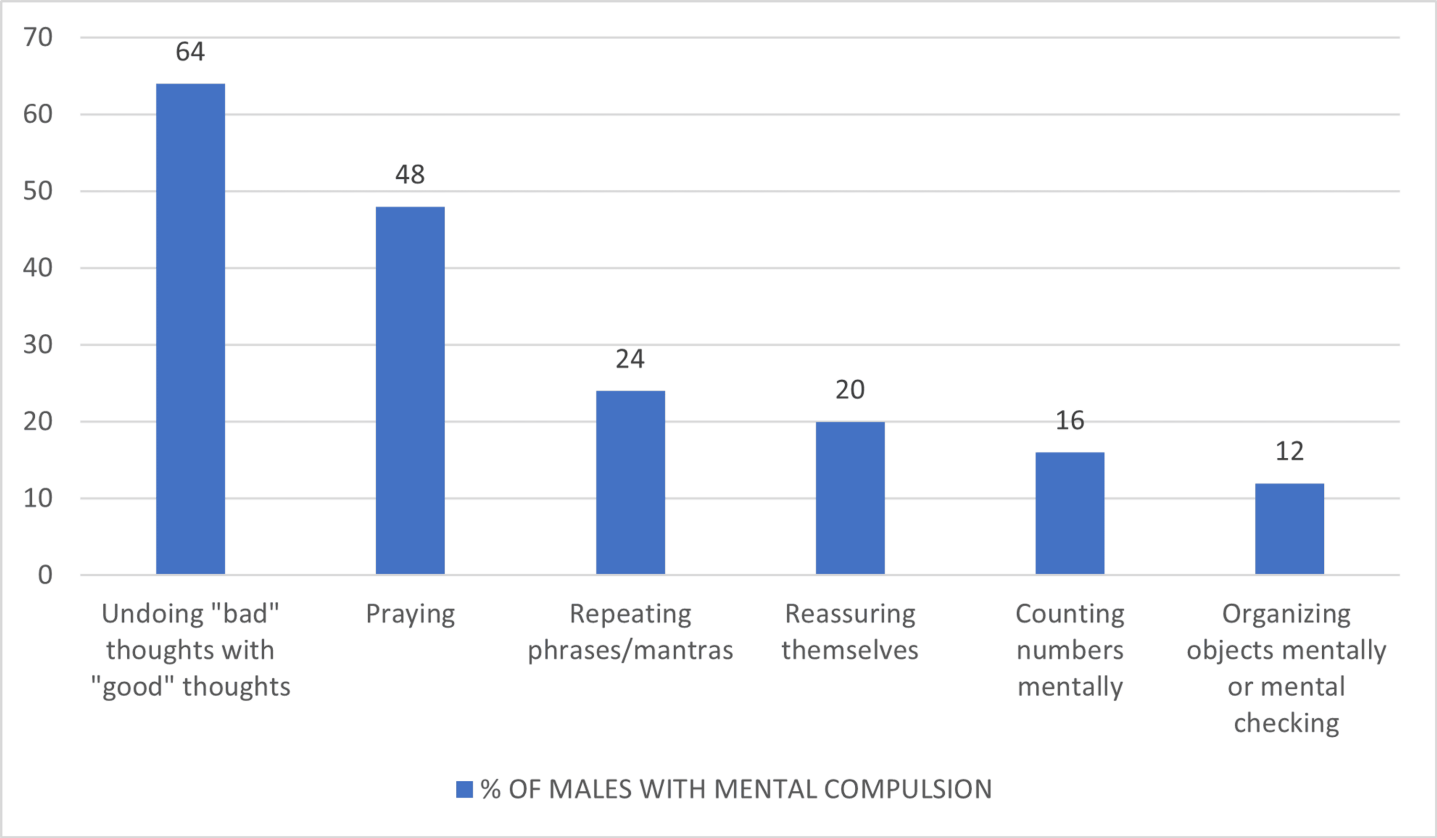
Types of mental compulsions in males

**Figure 4:**
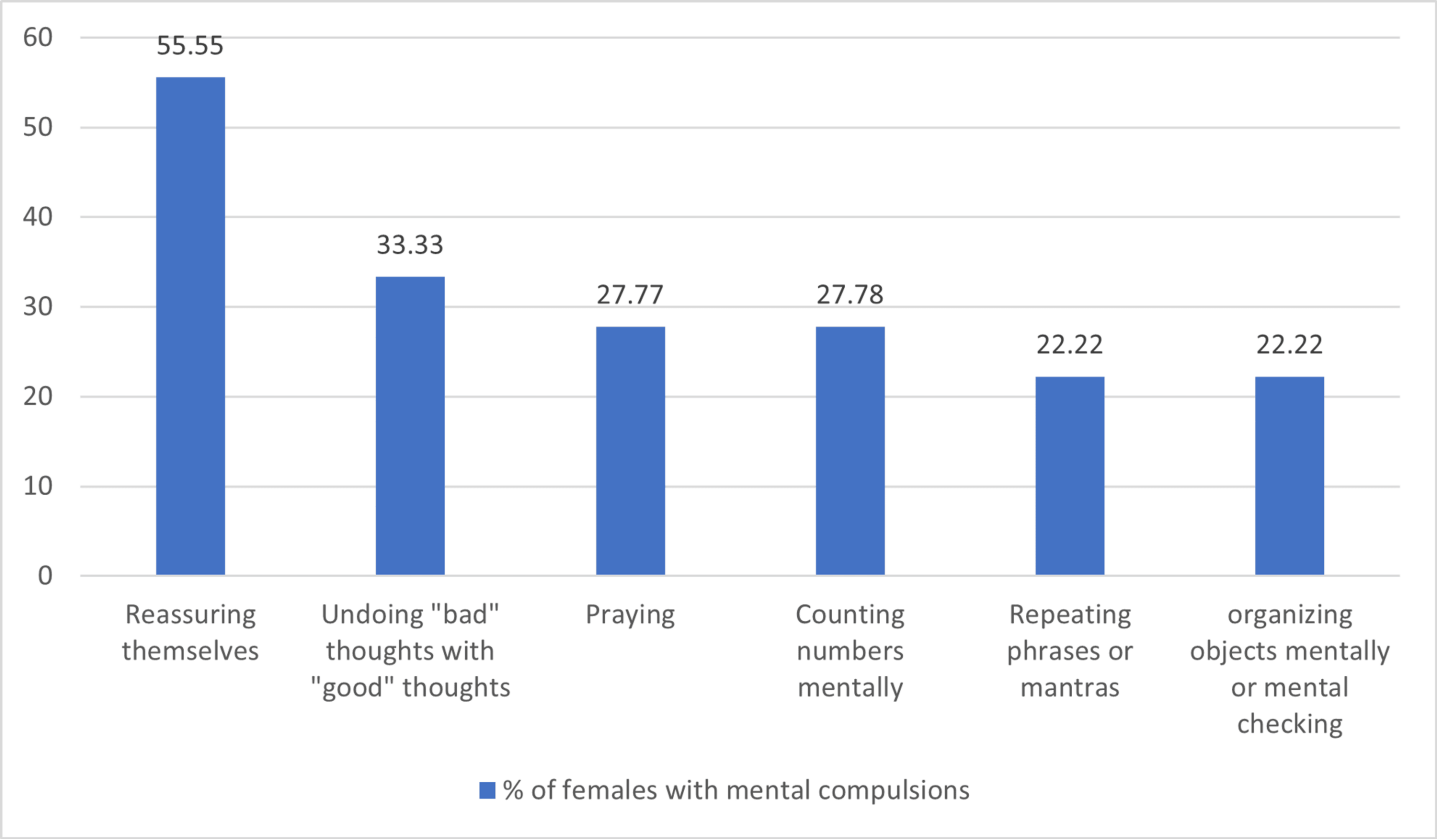
Types of mental compulsions in females

Among the 43 patients with mental compulsions, obsessions about sacrilege and blasphemy were the most common (35%), followed by obsessions related to symmetry (32%). The most common mental compulsions were undoing "bad" thoughts with "good" thoughts (51%), followed by praying (40%) and reassuring themselves (32%) (Figure 2). The most common mental compulsions in males were undoing "bad" thoughts with "good" thoughts (64%), followed by praying (48%) (Figure 3). The most common mental compulsions in females were reassuring themselves, followed by undoing "bad" thoughts with "good" thoughts (Figure 4).

**Figure 5:**
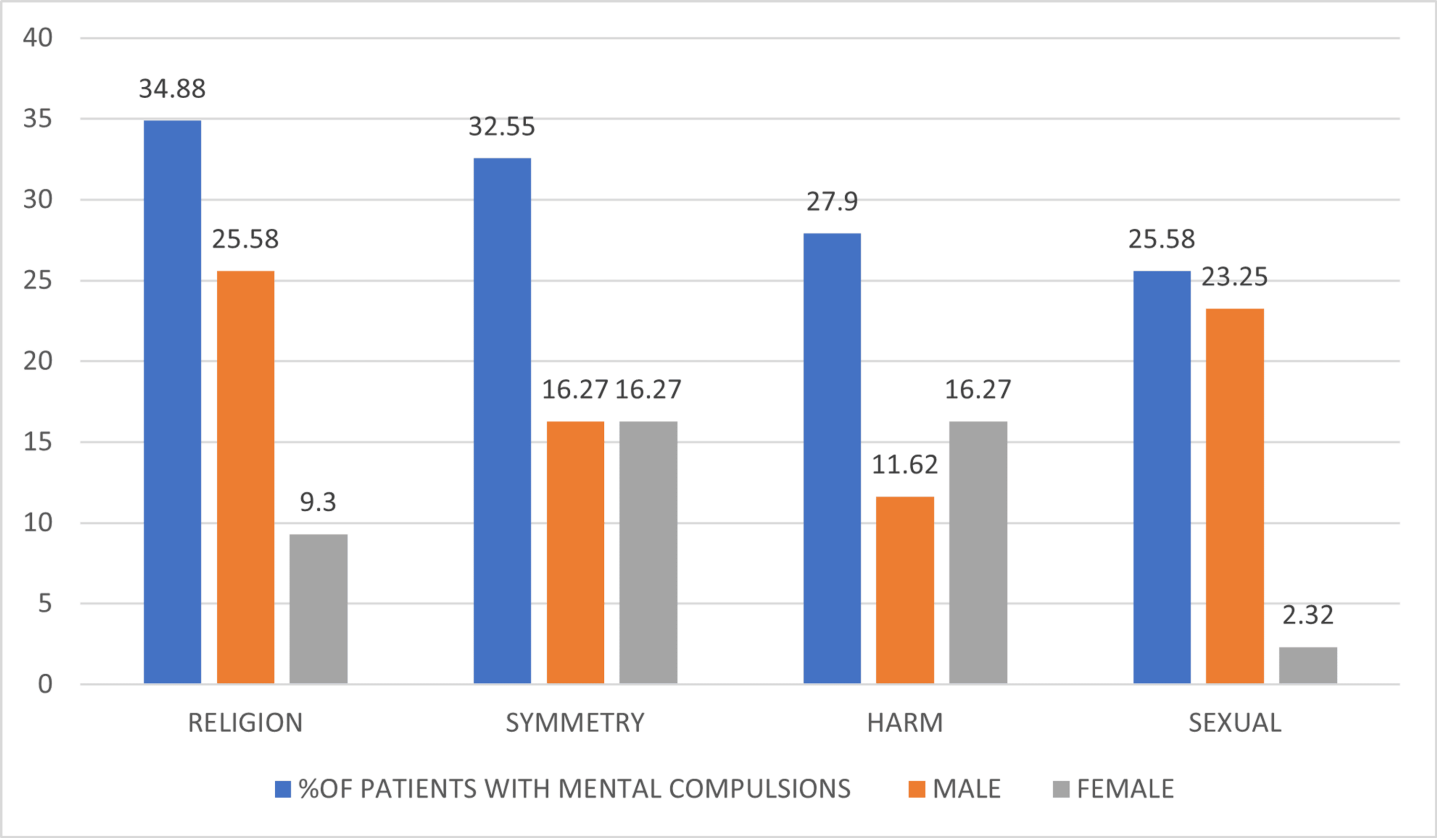
Content of obsessions secondary to which patients had mental compulsions

Most commonly, mental compulsions occurred following religious obsessions (34.88%), followed by symmetry (32.55%), harm (29.9%), and sexual obsessions (25.58%) (Figure 5). Additionally, 88% of patients with religious obsessions had mental compulsions. While 70.6% of patients have harm obsessions, 68.75% of patients have sexual obsessions, 50% of patients have symmetry obsessions, and only 5% of patients have dirt and contamination obsessions.

## Discussion

OCD was slightly more common in females than males in our study, and it was present in all socioeconomic classes, which is in accordance with prior epidemiological studies. According to a 2008 study by Ruscio et al. on the epidemiology of OCD in the National Comorbidity Survey Replication (NCS-R), OCD was found to be more common in women than in men [12]. The mean age of OCD onset in our sample was 22.3 years. OCD in females had a later onset than in males. Similar results were seen in the NCS-R study, which found that the odds of onset were highest from 18 to 29 years of age.

Using case data of individuals with OCD, a retrospective chart review conducted in 2022 by Srivastava and Agarwal revealed that the disorder began in the late 20s [13]. However, the quality and comprehensiveness of the analysis may be impacted by missing or inconsistent data in patient records because the study depends on retrospective chart checks. In a meta-analysis of OCD prevalence worldwide by Fawcett et al. in 2020 [14], which included 34 studies, it was found that women are more likely to experience OCD in their lifetime than men. Additionally, the study found that women had a lifetime prevalence of 1.5% against 1.0% in men. Additionally, compared to older adults, younger adults appear to have a higher lifetime risk of developing OCD, according to the study. Mathes et al. [15] conducted a general review in 2019 focusing on recent studies exploring gender variations in OCD. The review revealed that OCD appears to be more prevalent among males during childhood, but the prevalence shifts to females during adolescence and adulthood. Findings may be inconsistent due to variations in sample sizes, diagnostic standards, and data collection methods used in different studies. A large portion of the article's data comes from retrospective investigations, which are naturally constrained by recollection bias and the caliber of earlier medical records.

Additionally, males generally report an earlier age when OCD symptoms first appear compared to females. Jaisoorya et al. (2008) [16] examined gender differences in OCD and found that the average age of onset for OCD was earlier in males than in females. Males and females exhibited different predominant OCD symptoms; males more commonly reported having aggressive, sexual, and religious obsessions, and females tended to have more contamination obsessions and cleaning compulsions.

In 2007, Fontenelle et al. [17] conducted a qualitative comprehensive review of the literature, looking at studies on the genetic, environmental, and demographic risk variables linked to the start of OCD in epidemiological studies. Their review indicated that a substantial number of studies suggest late adolescence as a period of increased vulnerability for developing OCD. Furthermore, OCD was found to predominantly affect males during adolescence and females during adulthood. However, this study had limitations, such as a lack of consistent replication of findings and the presence of heterogeneity across studies.

Prevalence of mental compulsions

In the current investigation, the prevalence of mental compulsions was found to be 53.75%, which is comparable to the existing literature. In total, 58.13% of the participants endorsing mental compulsions were male, while 41.87% were female. Males had a higher prevalence of mental compulsions than females in our study, as mental compulsions were most common for religious or sexual obsessions, which were present predominantly in males. Similar results regarding the prevalence of mental compulsions in OCD were observed in a study conducted in 2023 by Ferrão et al. [4]. The study aimed to evaluate the psychopathological correlates and prevalence of mental compulsions in patients suffering from OCD. By comparing 447 patients without mental rituals with 519 patients who had mental rituals, the researchers found that the lifetime prevalence of mental rituals was 55.4%, and the current prevalence was 51.8%.

In 2017, Jaisoorya et al. [18] conducted a study to assess the prevalence of OCD and sub-threshold OCD among college students as well as their associations in Kerela, India. They found that 57.4% of students with OCD had mental compulsions. However, this study did not discuss the kind of mental compulsions or their severity. Similar findings were also observed in a multicentric investigation of the phenomenology of OCD by Shavitt et al. [3] in 2014. Using DY-BOCS, the study indicated that 56.7% of patients with OCD had mental compulsions.

In a 2011 longitudinal study by Sibrava et al., 12.9% of 225 patients with OCD reported mental compulsions as their primary compulsion [19]. Additionally, 20.4% indicated they were experiencing mental compulsions at the time, although not as a significant concern. Furthermore, 16.0% reported a history of mental compulsions but did not have them currently, and 50.7% had no history of mental compulsions. Most patients with mental compulsions also had behavioral compulsions. Similar results were seen in our study, as we observed that behavioral compulsions were present in most cases who endorsed mental compulsions. Only one case had mental compulsions without any behavioral compulsions.

Phenomenology of mental compulsions

There are only limited studies that have reported regarding the types of mental compulsions in patients with OCD. In this study, we found that the most common obsessions, overall, in patients with mental compulsions were about sacrilege and blasphemy (34.88%), followed by obsessions related to symmetry (32.55%), harm/aggression (27.0%), and sexual (25.58%). Most individuals had multiple mental compulsions (70%). The most common mental compulsions were undoing "bad" thoughts with "good" thoughts (51.2%), followed by praying (39.5%) and reassuring themselves (32.5%).

The most common obsessions in males with mental compulsions were about sacrilege and blasphemy, followed by sexual. The most common mental compulsions were undoing "bad" thoughts with "good" thoughts (64%) and praying (48%). The most common obsessions in females with mental compulsions were harm and symmetry, and the most common mental compulsions were reassuring themselves (55.5%) and undoing bad thoughts with good thoughts (33.33%).

Similar findings were observed in a study by Shavitt in 2014 [3], which found that mental compulsions were most common for symmetry dimension, e.g., organizing objects mentally; aggression, e.g., replacing aggressive thoughts with good thoughts, and the sexual or religious dimensions, e.g., thinking good thoughts after the thoughts that insulted God, repeatedly praying in the mind to relieve the anxiety caused by intrusive sexual thoughts. Our study also found that mental compulsions were most common, following sexual/religious dimensions and symmetry.

Sibrava et al. [19] conducted a study to evaluate the effects of primary mental rituals on clinical correlates, severity, and chronicity in OCD patients (N=225) over four years. They found that the most commonly endorsed obsession was over-responsibility for harm, followed by thoughts of sacrilegiousness and fears of offending God. Our study also found that the most frequently endorsed obsession was sacrilege and blasphemous thoughts, followed by symmetry and then harm. The most common compulsions were replacing "bad" with "good" thoughts followed by praying, which could be explained as religious and sexual obsessions were comparatively more common in this study.

Abramowitz et al. in 2003 [2] conducted two studies on 132 patients with OCD to assess mental compulsions and found that mental compulsions were most prevalent among patients with religious/blasphemous, aggressive, or sexual obsessions. These findings align with previous studies, which suggest that mental compulsions are more commonly associated with religious, aggressive, or sexual themes. Although the content of mental compulsions can vary due to social and cultural influences, the fundamental themes always stay the same. Because the sample is restricted to a particular area of India, it might not accurately reflect differences in OCD presentations or cultural influences on mental compulsions observed in larger or more diverse populations; hence, extensive national research may be required.

Limitations

The sample size for the study was minimal. Given that this study was carried out in a hospital, future community-based research might be done to determine the types and prevalence of mental compulsions seen in the general population. Also, the use of a semi-structured interview in our study instead of a structured interview (SCID) could potentially reduce diagnostic precision. We could not identify which came on first, bodily or mental compulsions. Furthermore, we did not evaluate how the symptom profile of mental compulsions changed with time. There was no evaluation of the treatment's effect on the mental compulsion. Before the commencement of OCD mental compulsion, no psychiatric diagnosis was evaluated.

## Conclusions

Mental compulsions were observed in over 50% of the study population. Mental compulsions were most common, following religious, symmetry, and harm-related obsessions. Most of the patients had multiple mental compulsions. The most common mental compulsions were undoing bad thoughts with good thoughts, followed by praying, reassuring themselves, and repeating phrases or mantras. Given the high prevalence of mental compulsions in OCD, further longitudinal studies are needed to assess their impact on the treatment outcome. Future research could also focus on developing behavioral therapies that deal with this particular symptom dimension in OCD.
